# Biodegradable elastic nanofibrous platforms with integrated flexible heaters for on-demand drug delivery

**DOI:** 10.1038/s41598-017-04749-8

**Published:** 2017-08-23

**Authors:** Ali Tamayol, Alireza Hassani Najafabadi, Pooria Mostafalu, Ali K. Yetisen, Mattia Commotto, Musab Aldhahri, Mohamed Shaaban Abdel-wahab, Zeynab Izadi Najafabadi, Shahrzad Latifi, Mohsen Akbari, Nasim Annabi, Seok Hyun Yun, Adnan Memic, Mehmet R. Dokmeci, Ali Khademhosseini

**Affiliations:** 1 Biomaterials Innovation Research Center (BIRC), Division of Engineering in Medicine, Brigham and Women’s Hospital, Harvard Medical School, Boston, MA 02139 USA; 2 Harvard-MIT Division of Health Sciences and Technology, Massachusetts Institute of Technology, Cambridge, MA 02139 USA; 3 Wyss Institute for Biologically Inspired Engineering, Harvard University, Boston, MA 02115 USA; 4 Department of Pharmaceutical Sciences, University of Michigan, Ann Arbor, MI 48109 USA; 5 Harvard Medical School and Wellman Center for Photomedicine, Massachusetts General Hospital, Cambridge, MA 02139 USA; 6 Center of Nanotechnology, King Abdulaziz University, Jeddah, 21569 Saudi Arabia; 7 Department of Biochemistry, King Abdulaziz University, Jeddah, 21569 Saudi Arabia; 8 Washtenaw Community College, Ann Arbor, MI 48105 USA; 9 Department of Neurology, David Geffen School of Medicine, University of California Los Angeles, Los Angeles, California USA; 10 Department of Mechanical Engineering, University of Victoria, Victoria, BC Canada; 11 Department of Chemical Engineering, Northeastern University, Boston, MA 02115-5000 USA; 12 Department of Bioindustrial Technologies, College of Animal Bioscience and Technology, Konkuk University, Seoul, Republic of Korea

## Abstract

Delivery of drugs with controlled temporal profiles is essential for wound treatment and regenerative medicine applications. For example, bacterial infection is a key challenge in the treatment of chronic and deep wounds. Current treatment strategies are based on systemic administration of high doses of antibiotics, which result in side effects and drug resistance. On-demand delivery of drugs with controlled temporal profile is highly desirable. Here, we have developed thermally controllable, antibiotic-releasing nanofibrous sheets. Poly(glycerol sebacate)- poly(caprolactone) (PGS-PCL) blends were electrospun to form elastic polymeric sheets with fiber diameters ranging from 350 to 1100 nm and substrates with a tensile modulus of approximately 4-8 MPa. A bioresorbable metallic heater was patterned directly on the nanofibrous substrate for applying thermal stimulation to release antibiotics on-demand. *In vitro* studies confirmed the platform’s biocompatibility and biodegradability. The released antibiotics were potent against tested bacterial strains. These results may pave the path toward developing electronically controllable wound dressings that can deliver drugs with desired temporal patterns.

## Introduction

In many regenerative processes, different bioactive cues are involved at various stages of healing. One prominent challenge in many reconstructive and regenerative therapies is controlling bacterial infection, in particular in the treatment of chronic wounds and burns^1^. The skin is the primary protective barrier against environmental pathogens and if damaged bacterial risk factors increase significantly^2–4^. Some strains of pathogenic bacteria such as *Pseudomonas aeruginosa* and *Staphylococcus aureus* are detrimental to wound healing and if not properly managed can significantly increase the morbidity and mortality rate^5, 6^. Similarly, despite safety measures the rate of failure in implantation of surgical meshes due to infection is significant^7^.

Existing infection treatment strategies include oral and intravenous administration of antibiotics that can reach up to 2000 mg per dosage^8^. Such routes of administration can have side effects on healthy tissues or organs and are of significant concern due to formation of antibiotic resistance^9^. One promising strategy for management of bacterial infection is based on localized delivery of antibiotics at a precise dosage only when necessary^10^. For instance, it was shown that the localized therapy resulted in fewer surgical site infections after dermatological surgery as compared to patients administered perioperative oral antibiotics^8^. In recent years, advances in drug delivery platforms and wound dressings have enabled the passive controlled release of active compounds^11–14^. For example, several wound dressings have been developed that use antibiotics or antiseptics (*e.g*., silver or iodine)^15–17^. While such platforms and wound dressings demonstrated sustained release of antibiotics, they often provide limited controllability on the release rate of drugs^18, 19^. Antiseptic agents in wound dressings may impair functions necessary for wound healing, thus they only should be released if necessary^20^. However, existing wound dressings are usually not capable of releasing these compounds on-demand. Thus, there is a clear need for advanced meshes that can be used in both implantable and topical applications for on-demand release of bioactive compounds including antibiotics. One possible solution for achieving this goal is to employ stimuli-responsive polymers which can respond to external stimulus for active drug release.

**Figure 1:**
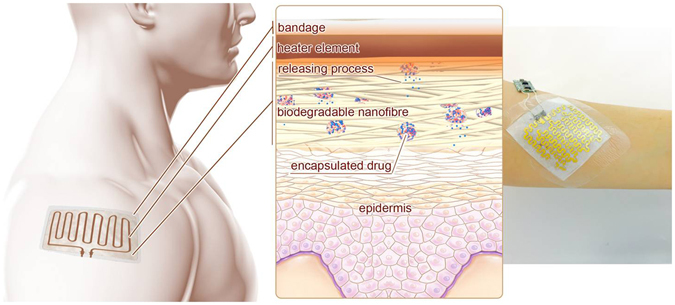
The principle of operation of the drug delivery system and a typical fabricated bandage with integrated heater and electronics. Thermo-responsive drug nanocarriers were embedded within nanofibers of the engineered mesh and released their payload upon temperature increase induced by the integrated flexible heater. A typical fabricated bandage with the miniaturized control electronics is shown on the right.

The fabrication of flexible electronics on non-conventional platforms has enabled the realization of systems that form conformal contact with non-flat surfaces (*i.e*., skin)^21–25^. Thus, integrated electrical systems can be used as a promising platform for applying the external stimulation required for triggering drug release^19, 26, 27^. For instance, electrical heaters can be powered via wires or wirelessly to generate heat as a safe external stimulation method. Here, we create an elastic nanofibrous mesh made of PGS-PCL containing PEGylated-chitosan based thermo-responsive drug nanocarriers. An integrated flexible heater was used to generate heat on-demand to trigger the drug release (Fig. 1). The heater was fabricated from various metals that can render the platform with improved biocompatiblilty and can be made fully biodegradable if required. The platform was used for controlled release of various antibiotics such as cefazolin and ceftriaxone encapsulated in nanoparticles. A microcontroller was used to tune the voltage throughout the conductive film to apply heat to the wound dressing. Upon stimulation by the heating unit, these thermo-responsive nanoparticles released encapsulated antibiotics at a certain range of temperature (38–40 °C). This platform can open the way toward the realization of fully degradable smart surgical meshes.

## Results and Discussion

**Figure 2:**
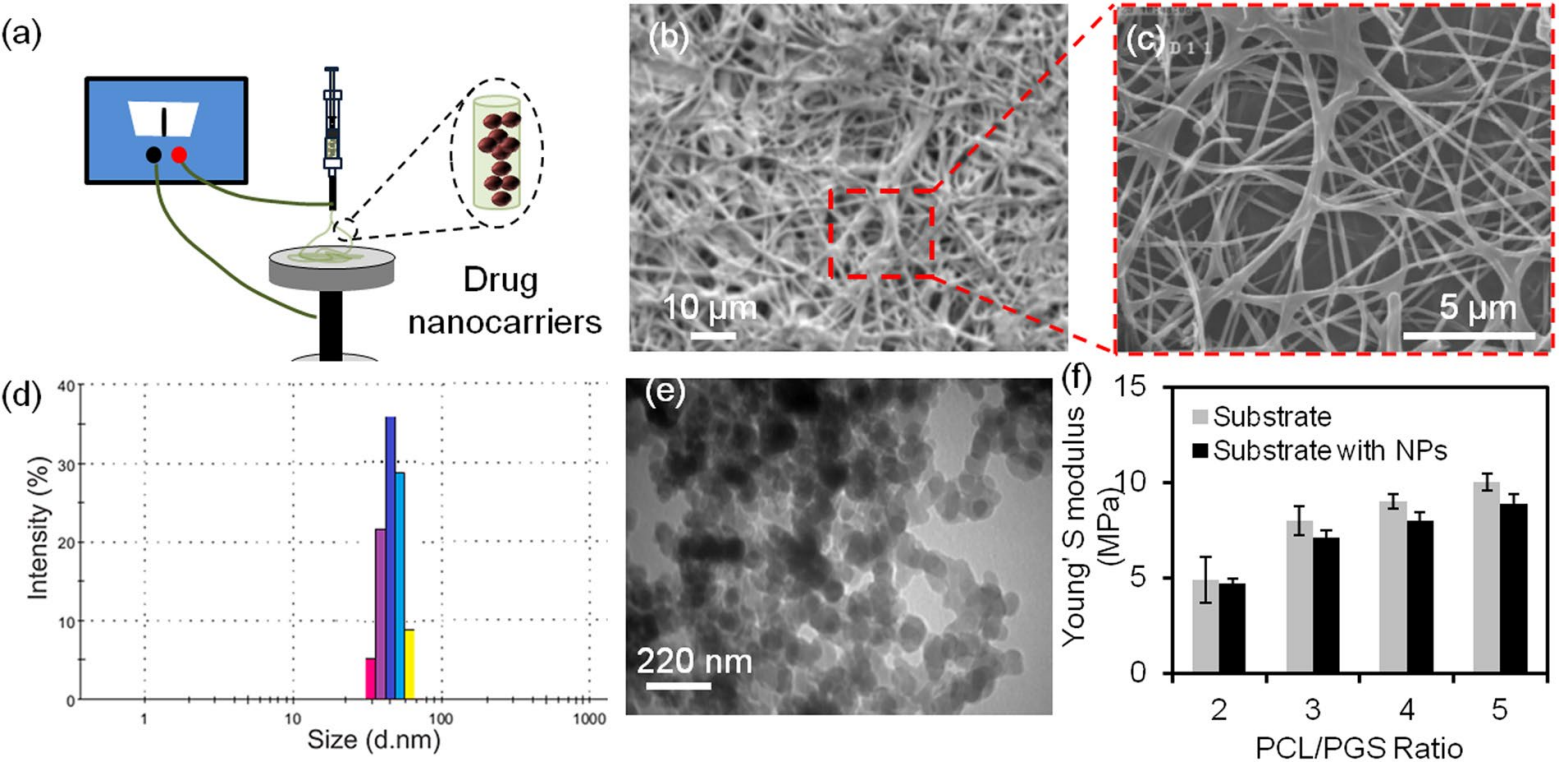
Fabrication of the elastic biodegradable nanofibrous drug delivery platform. (**a**) Schematic of the fabrication of the substrate by electrospinning (**b**,**c**) Representative SEM images of a typical nanofibrous substrate containing drug nanocarriers. (**d**) Size distribution of the generated particles in wet conditions measured by DLS. (**e**) TEM image of PEGylated chitosan nanoparticles which represented the spherical shape of nanoparticles and particles size. (**f**) Effects of nanocarriers on the Young’s modulus of the nanofibrous substrate containing 10% (w/w) of nanocarriers.

The goal of this study was to develop a mesh-like platform that could be used in both internal and topical applications and could release drugs on-demand. In addition to biocompatibility and tunable degradability, such a platform should have flexibility and elasticity comparable to native tissues. Recently, we have developed nanofibrous substrates for the fabrication of elastic electronics^28^. In addition to suturability, these nanofibrous meshes offer high surface area-to-volume ratio, which is important for effective drug release^29^. Thus, we fabricated PGS-PCL nanofibrous membranes using electrospinning (Fig. 2a–c). These meshes were highly stretchable and possessed extensibility of over 100% of their initial length (Figure S1).

To achieve on-demand drug release, thermo-responsive nanoparticles were synthesized from a PEGylated-chitosan blend. PEGylated-chitosan is biodegradable, biocompatible, non-toxic and immunocompatible; thus, it has been widely used in pharmaceutical fields^30–32^. PEGylated-chitosan has a controlled transition temperature and antibacterial characteristics as compared to other thermo-sensitive polymers^31^. This material goes through a sol-gel transition at the critical temperature of ~37 °C (Figure S2); details of material characterization is provided in Supplementary Information (Figures S2, S3). This temperature is slightly higher than the skin temperature (32–36 °C)^33^.

Nanoparticles were fabricated by the emulsification of aqueous PEGylated-chitosan solution (1.0% w/v; 1 mL) in methylene chloride (1 mL) and genipin solution (5 mL of 0.05 mM) to rapidly form nanoparticles without agglomeration. The nanoparticle size distribution and morphology were determined using transmission electron microscopy (TEM), dynamic light scattering (DLS), as well as atomic force microscopy (AFM). The nanoparticles had a mean diameter of 80 nm in solution with a narrow size distribution (Fig. 2d). The TEM images also confirmed the uniform size distribution determined using DLS (Fig. 2e). The difference in the DLS data could be due to the swelling of drug carriers as the DLS measurements were performed in aqueous environment as compared to TEM imaging which were conducted at dry state. It is known that the hydrogel-based particles possess high water uptake and rapid swelling, thus, the hydrated particles used in the DLS system were swollen^31, 34, 35^. In the present study, all the measurements were carried out when the equilibrium state was reached. TEM images indicated that the particles were spherical. These measurements were further verified by AFM, which showed uniform size distribution without aggregation (Figure S4). This is an important factor in achieving reproducible and predictable release profiles. The advantage of AFM data is that the particles are neither at the swollen state nor under vacuum. Thus, the obtained results are the closest to the actual working condition of the particles encapsulated within nanofibers. The size distribution of the particles obtained from AFM and TEM images are also shown in Figure S4. The approximate size of the particles measured by TEM was around 60 nm, while the value for the images obtained from AFM was around 70 nm.

Nanofibrous meshes were fabricated by electrospinning of PGS-PCL prepolymer solution (20%, w/v) prepared at different ratios. The mechanical properties of the electrospun meshes such as their elastic modulus were tuned by changing the PGS:PCL ratio (Fig. 2f). In addition, PEGylated-chitosan nanoparticles were mixed with the prepolymer solution at the concentration of (15%, w/v) and were electrospun. The mechanical strength and stiffness of hydrogels have been reported in the literature and are significantly lower than the polymeric substrate^36^. Thus, when PEGylated-chitosan particles were added to the polymeric network, weak points would have been generated throughout the polymeric network. Thus, the overall mechanical properties of the substrate could be reduced due to the presence of the nanocarriers. However, the incorporation of the nanoparticles within the size range used did not significantly change the elastic modulus of the nanofibrous mesh (Fig. 2f).

**Figure 3:**
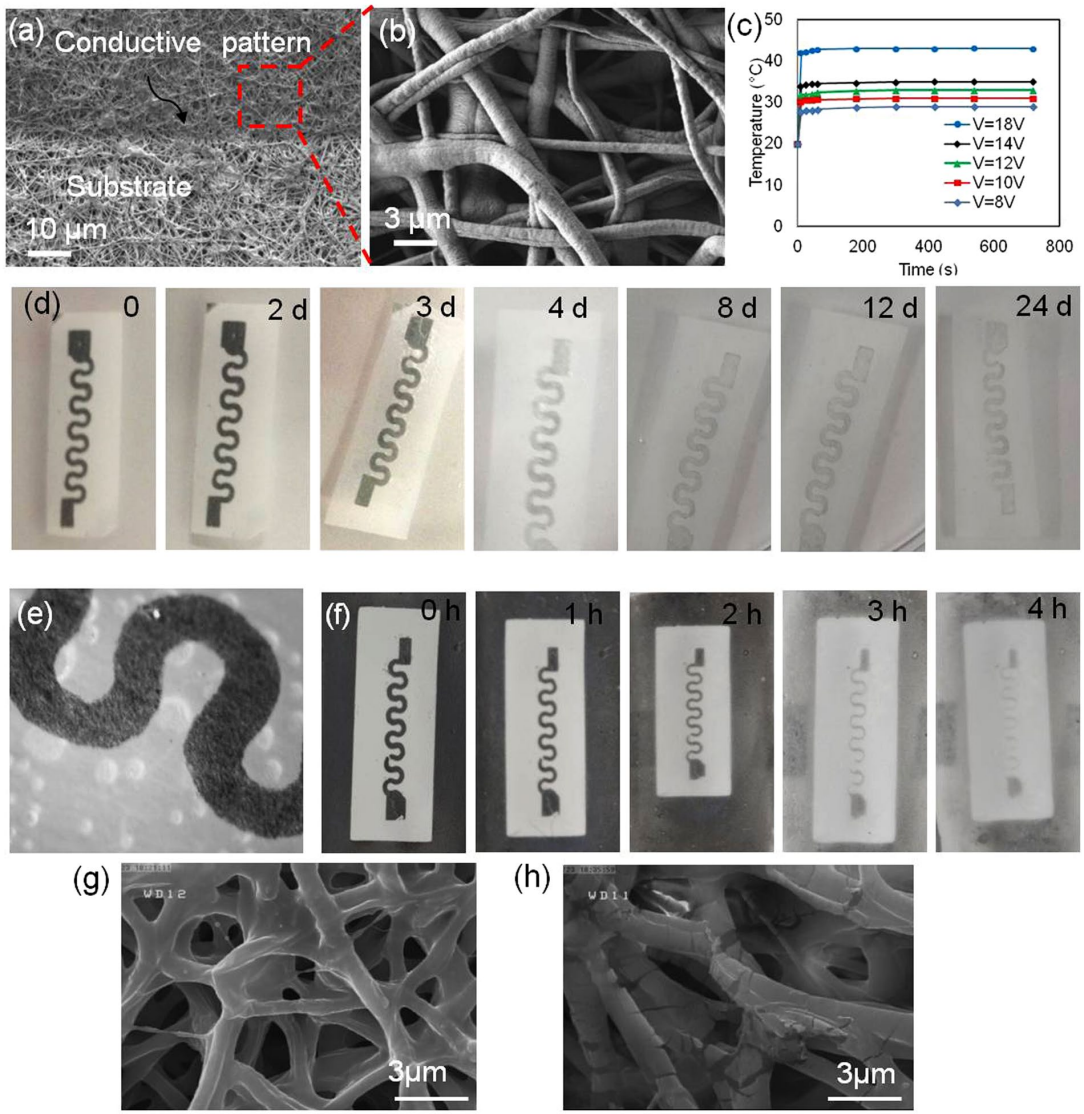
Characterization of the nanofibrous substrate containing nanocarriers and the engineered heater. (**a**,**b**) Representative images of a nanofibrous substrate with a biodegradable Zn heater deposited by RF sputtering. (**c**) Characterization of the zinc microheater, the resulting temperature as a function of applied voltage. (**d**) Degradation of zinc patterns over 24 days in aqueous solutions containing NaOH (2.5 mM). (**e**) Micrograph showing the hydrolysis of zinc patterns over 24 days. (**f**) Degradation of magnesium patterns over 4 h in aqueous solutions containing 2.5 mM NaOH. (**g**) A representative SEM image of a nanofibrous substrate heated to 38 °C for 1 h confirming the stability of the membranes at that temperature. (**h**) A representative SEM image of nanofibrous substrate heated to 50 °C for 1 h, showing melted, decomposed and deformed state.

We have previously fabricated electrical circuitry and conductive patterns on nanofibrous meshes using screen printing and inkjet printing of conductive inks made of silver particles and carbon nanotubes^25, 28^. However, the low pattern substrate fidelity, toxicity and immune response against the materials used in development of these inks were a key limitation in their biomedical applications. In the present work, we used low temperature radio frequency (RF) sputtering that allowed the deposition of various metals onto the substrate. The RF sputtering was performed on the nanofibrous substrate, mounted onto a cooled sample holder, which was able to control the temperature of the sample during the deposition process. The temperature of the samples was kept lower than 25 °C during deposition, which was below the critical temperature of PEGylated-chitosan particles. Using this technique, we generated conductive patterns from various metals including silver, gold, zinc, and magnesium. The utilized deposition method allowed the penetration of metal into the substrate that improved pattern-substrate fidelity (Fig. 3a,b). We did not observe the detachment of the pattern from the substrate in SEM imaging. We also assessed the possibility of sustaining a stable temperature using heat generated by applying electrical voltage to fabricated patterns and measuring their surface temperature (Fig. 3c). It can be seen that the surface temperature was stable for up to 10 min. The fabricated conductive patterns were stretchable. Figure S5 shows the variation of the electrical resistance of a typical conductive pattern sputtered on a nanofibrous substrate versus the applied strain. The conductive patterns maintained a linear increase in resistance versus strain behavior for up to 20% strain.

Although degradability of PGS-PCL as a substrate for flexible electronics has been previously demonstrated, the fabricated conductive patterns were not resorbable in those cases^28^. The ability to generate patterns from zinc and magnesium enabled the present construct to be a fully degradable platform. To test the degradability of the engineered conductive patterns, an alkaline solution (2.5 mM of NaOH in PBS) was used. Alkaline solutions enhance the degradation rate and enable the investigation of degradability of the engineered constructs. Since the pH of the chronic wound environment is less than 9^37–39^, we expect that the degradation rate of the conductive patterns to be similar. Determining the exact degradation profile at various pH values requires comprehensive experiments which are beyond the scope of the present study. However, our experiments suggested a slow degradation of zinc patterns at pH of 8.5 (Figure S6). The hydrolysis of zinc patterns and resulting degradation can be seen in the micrograph shown in Fig. 3d. The degradation rate of the patterns was material dependent. For instance, zinc patterns were present for over 20 days, while magnesium patterns degraded in less than one day (Fig. 3d–f). Electrical heaters were fabricated on the nanofibrous meshes as described above and voltage applied to the heater was varied to alter the mesh temperature. SEM images were taken from heated nanofibrous meshes to study the effect of temperature on the stability of nanofibrous meshes (Fig. 3g,h). Slight defects were observed in the fibers that were heated up to 38 °C (Fig. 3g), while the morphology of the nanofibers heated up to 50 °C was changed (Fig. 3h). The slight deformation in the microstructure might be due to swelling and deformation of the incorporated nanoparticles. Therefore, we expect that the stability of the fibers at temperatures close to body temperature does not allow sudden release of drug or nanoparticles from the mesh.

We evaluated the effectiveness of the platform for encapsulation and on-demand release of commercial antibiotics including cefazolin and ceftriaxone. Drug encapsulation efficiency of PEGylated-chitosan were investigated at different concentrations of drug (0.1–3.0% w/v) and polymer (0.1–1.0% w/v) (Tables S1, S2). Results indicated that increasing the encapsulation efficiency of ceftriaxone was a function of PEGylated-chitosan concentration in the solution to a maximum of 91% at 1% (w/v). A non-linear relationship was observed between the ratio of drug and PEGylated-chitosan concentrations and encapsulation efficiency. We observed the best encapsulation efficiency at ratio of 3:1 (w/w) drug to PEGyelated-chitosan. The encapsulation efficiency was ~83% by addition of 3% (w/v) of cefazolin and ~91% for 3% (w/v) of ceftriaxone. The encapsulation efficiency decreased to 75% and 68% for 3.5% (w/v) aqueous solutions of cefazolin and ceftriaxone, respectively. This difference was perhaps due to the difference in the interaction between drugs and the polymer. Thus, maximum 3% (w/v) drug concentration was used throughout the experiments.

**Figure 4:**
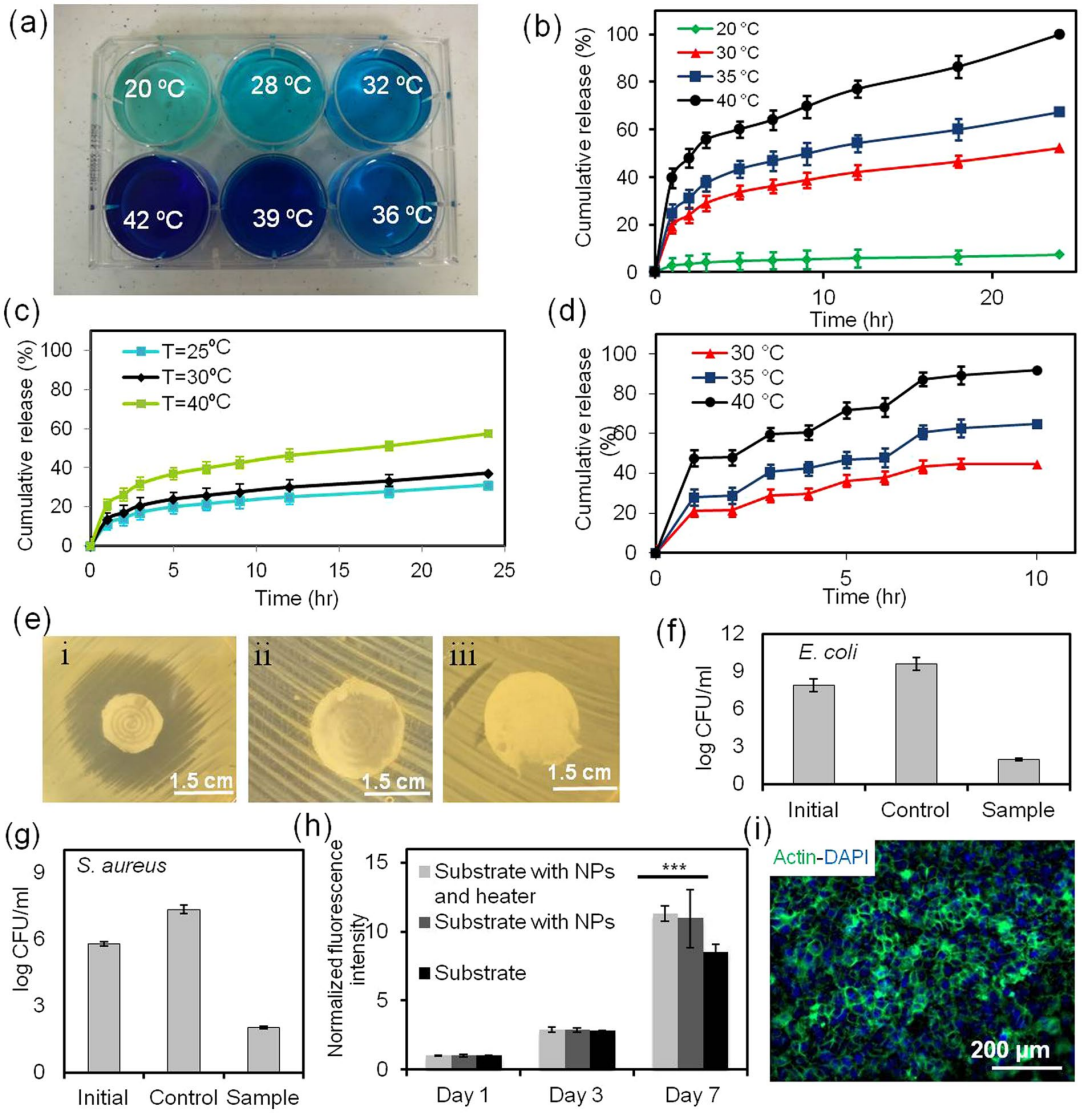
Characterization of the release profile and biocompatibility of the engineered platform. (**a**) Effect of temperature on the intensity of the methylene blue release from the nanofibrous platform. (**b**) The spectrum of release rate of cefazolin from the nanofibrous drug delivery system at different temperatures. (**c**) Effect of temperature on the interfacial release rate of ceftriaxone from the nanofibrous drug delivery system. (**d**) Cyclic thermal stimulation at different temperatures for on-demand release of cefazolin. (**e**) Assessment of the effectiveness of the released drug against bacterial culture; occurrence of a clear inhibition zone around the mesh containing cefazolin at 38 °C (i) in comparison to the mesh without cefazolin (ii) and the mesh containing cefazolin without heater after 12 h (iii). (**f**,**g**) Effect of the released antibiotics on the number of CFUs in a bacteria solution after 24 h of culture; ceftriaxone against *E. coli* and cefazoline against *S. areus*. (**h**) Metabolic activity of human keratinocytes cultured in contact with the nanofibrous substrate with and without nanoparticles and the flexible heater (***p < 0.001). (**i**) Immunostaining against F-actin and cell nuclei (DAPI) confirming the normal morphology of the cells cultured in contact with the nanofibrous substrate.

The capability of PEGylated-chitosan loaded PGS-PCL nanofibrous mesh to release drugs by different rates was assessed at different temperatures. Drug loaded nanoparticles were mixed with the PGS-PCL prepolymer solution (15% w/v) prior to electrospinning. To visualize the release from the nanofibrous meshes, we encapsulated methylene blue into nanoparticles prior to the fabrication of electrospun meshes. The meshes were placed into different wells of a multiwell plate containing phosphate buffered saline (PBS) and different voltages were applied to create different surfaces temperatures. The volume of the solution during the test was kept constant and the evaporated solution was adjusted every 8–12 h by adding fresh solution. In addition, to prevent excessive evaporation the containers were covered with an impermeable lid. As shown in Fig. 4a, increasing the surface temperature increased the release rate of methylene blue from nanofibers, which in turn enhanced the color intensity of the surrounding liquid.

Similarly we assessed the release rate of cefazolin and ceftriaxone from the nanofibrous system. The solubility of the drug can potentially change by variations in temperature. However, the tested drugs are hydrophilic and are highly water soluble. Therefore, at the concentrations used, the solubility was not an issue and did not affect the release rate. Figure 4b,c indicates that about 40% of cefazolin was released from nanofibers during the first 2 h, and around 98% of cefazolin was released after 24 h at 40 °C. However, for T = 20 °C less than 8% of cefazolin was released from nanofibers after 24 h, which could be attributed to transition temperature of the nanoparticles (T_cr_~ 37 °C). In addition, no initial burst release was observed for samples and controls. The drug release profile of ceftriaxone was generally similar to cefazolin, but the difference between the concentration of the drug released over same temperature and time for both drugs could be attributed to the size of drug molecules and interaction between drug molecules and polymers. Since the transition temperature of the PEGylated-chitosan is ~37 °C, the sol to gel transition starts around 30 °C, which initiates the drug release and becomes completed ~ 40 °C (Figure S3). We also investigated the possibility of prohibiting the drug release on demand. Heat was applied in pulses/periods of 1 h while the heat application was stopped for another 1 hr. The drug release was stopped once the surface temperature was reduced (Fig. 4d). Thus, the nanofibrous mesh can be used for the delivery of antibiotics and drugs with a desired temporal pattern. Since the human skin temperature is around 33 °C, the fabricated platform with a critical temperature of 37 °C can be implemented for topical applications. Thus, to prevent uncontrolled release of encapsulated drugs due to external temperature changes in topical applications, the platform should be thermally insulated. However, for internal application, the synthesized thermo-responsive polymer could be co-polymerized with other biocompatible monomers to increase its critical temperature. We also assessed the functionality and effectiveness of the released drug after thermal stimulation. Nanofibrous meshes containing nanoparticles with and without cefazolin were fabricated as described before. To evaluate the effectiveness of the released antibiotics, zone of inhibition and number of colony forming units (CFU) methods were employed. Gram positive and Gram negative bacteria such as *S. aureus* and *E. coli* were cultured on the agar plates and samples of ~1.5 cm in diameter were placed on the cultured plates. The surface temperature was increased to 38 °C using the integrated flexible heaters and the zone of inhibition was determined after 24 h of culture (Fig. 4e). The sample containing antibiotics had a noticeable zone of inhibition (~10 mm), which was not observed in the heated sample without antibiotics and the sample containing antibiotics without a heater (Fig. 4e). We also assessed the number of CFUs in a bacterial culture. Circular samples (15 mm in diameter) were placed in a bacterial culture (10 mL) and were heated at a constant rate to increase the surface temperature to 38 °C. After 24 h of culture, the number of CFUs was significantly reduced (Fig. 4f,g and Table S4).

The biocompatibity of the engineered nanofibrous meshes was assessed by culturing human keratinocytes on the meshes and monitoring their metabolic activity and morphology. The metabolic activity of cells cultured on the surface of the mesh containing thermo-sensitive particles and drug carriers was measured using Presto Blue assay. No significant difference was observed between the cells cultured on meshes with and without nanoparticles. However, the meshes that included silver-based heaters showed lower metabolic activity (Fig. 4h). This reduction was possibly due to the inhibitory effect of silver on keratinocytes growth^40^. Staining against F-actin showed normal cellular morphology on the electrospun meshes (Fig. 4i).

Nanofibrous meshes have been used as scaffolds for skin growth in treating burns and wounds. The mechanical properties of PGS-PCL nanofibrous meshes including their elasticity and elastic modulus are similar to the values for skin^28^. Thus, in addition to topical applications as shown in Fig. 1, the suturability of the engineered nanofibrous mesh allows its use as a scaffold for cell growth as well as closing deep wounds.

## Conclusions

A nanofibrous elastic mesh was engineered to carry stimuli-responsive drug carriers and could be used as a substrate for the fabrication of flexible heaters. Thermo-responsive particles of PEGylated-chitosan were fabricated with a critical temperature ~37 °C. The nanoparticles were embedded within the nanofibrous meshes and were loaded with antibiotics. A flexible heater was integrated into the platform to allow on-demand release of drugs. The engineered platform was flexible, elastic and could form conformal contact with native tissues. The platform could be either used as a topical patch or possibly as an implantable scaffold for wound closure. In the latter case, heaters could be fabricated from biocompatible and bioresorbable metals including zinc and magnesium, where their degradation rate could be tuned. The drug release rate was proportional to the applied voltage that generated heat and altered the surface temperature. The effectiveness of released antibiotics was assessed against the culture of different bacterial strains inhibiting their growth. The engineered platform can be further developed for engineering smart surgical meshes, tissue engineering scaffolds, and wound dressings, which can release drugs in response to external stimuli on-demand. Also, other stimuli-responsive materials can be incorporated into the platform to allow independent release of multiple drugs.

## Experimental Section

### Materials

Chitosan, medium viscosities of 200–800 cps, acetic acid, poly(ethylene glycol) monomethyl ether (mPEG) (MW 2000), sodium dodecylsulfate (SDS), anhydrous tetrahydrofuran (THF), genipin (GP), sodium hydroxide (NaOH), anhydrous *N*,*N*-dimethylformamide (DMF), oxalyl chloride, poly ɛ-caprolactone (PCL) with average molecular weight 80000), glacial acetic acid, sodium hydride (NaH), anhydrous pyridine, acetonitrile, and methanol were purchased from Sigma-Aldrich (St. Louis, MO, USA). Anhydrous chloroform and ethanol were purchased from Fisher Scientific (USA). Cell culture reagents including Dulbecco’s modified Eagle medium (DMEM), 0.05% trypsin-EDTA (1X), fetal bovine serum (FBS), and Penicillin/Streptomycin were purchased from Invitrogen (Carlsbad, CA, USA). PrestoBlue^®^ Assay and Live/Dead Cell Viability Kit were obtained from Life Technologies. Cefazolin sodium salt (≥98% purity) was obtained from Fluka (Buchs, Switzerland).

### Synthesis of PGS

Synthesis of PGS (MW 12,000) was performed as described before^41^. Briefly, PGS was prepared by polycondensation of sebacic acid and glycerol under inert gas atmosphere at 120 °C for at least 24 h. The pressure was reduced to 40 mtorr over 48 h to prepare PGS.

### Preparation of PEGylated-chitosan

Chitosan grafted poly(ethylene glycol) monomethyl ether (mPEG) was prepared as described previously^42, 43^. Briefly, sodium dodecylsulfate (SDS) solution in water was mixed with the chitosan and the suspension mixed for 2 h at room temperature. The precipitation was filtered off, washed with DI water three times, and freeze-dried to obtain SDS/chitosan. Then, oxalyl chloride was mixed with 10-fold of SDS/chitosan weight (0.1 g) in anhydrous pyridine (50 mL). The reaction was continued at room temperature over 30 min under pre dried inert gas atmosphere. Consequently, the products were cooled down and freeze dried to extract chlorinated-SDS/chitosan. Then, mPEG (mPEG, 4 g) was activated by adding NaH (10 mg) into 50 mL of THF solution to form OH-PEG-OCH_3_. The reaction was carried out under continuous stirring at 60 °C for 2 h under inert gas atmosphere. Then, prepared chlorinated-SDS/chitosan (60 mg) was added slowly to the reaction and mixed for one day, under the same condition. Finally, the products were cooled down to room temperature and precipitated in methanol. The percipients were then washed and freeze dried. The SDS protection was removed by adding 15% (w/v) tris (hydroxyl methyl) amino methane aqueous solution and adjusting the pH to 8 using HCl (5.0 M) at room temperature. The precipitates were then freeze-dried to obtain chitosan-graft-PEG (PEGylated chitosan).

### Characterization of polymer synthesis

Proton-nuclear magnetic resonance (1 H NMR) was conducted on Bruker Avance 800 MHz NMR spectrometer to analyze the chemical structure and degree of substitution on mPEG to chitosan. Briefly, 10–20 mg of each sample was dissolved in deuterium oxide (D_2_O) containing 0.5 M DCl/D_2_O or dimethyl sulfoxide (DMSO) as a solvent. ^1^H-nmr data of chitosan and PEGylated chitosan was obtained as follows: the chemical shifts of chitosan and PEGylated chitosan are: 1.85 (s, 3 H, -C(O)OCH_3_), 2.57 (s, 3H-CH3), 3.59 (s, 1 H, H2), 3.97–4.12 (m, 4 H, H5), 4.14–4.51 (m, 5 H, H6, H3, H4), 5.30 (s, 1 H, H1). PEGylated chitosan: 1.85(s, 3 H, -CH_3_ in acetamide of chitosan), 2.85 (s, 1 H, H2 in chitosan), 3.3 (s, 3 H, -OCH_3_), 3.2–3.8 ppm (m, nH, PEG-OCH_2_CH_2_O and, H3 and H6 in chitosan (pyranose ring).

### Fourier Transform Infrared Spectroscopy (FT-IR)

Fourier transform infrared spectroscopy (FT-IR) was carried out using a spectrometer (Shimadzu 8400 S FTIR, Osaka, Japan). For FTIR spectra analysis, a dried sample (10 mg) was mixed with dry KBr (500 mg). The mixture was pressed to form disks using a macro KBr die kit. The solid pellet was scanned at 4 cm^−1^ in FTIR instrument.

### Nanoparticle preparation

Nanoparticles were prepared using emulsification ionic gelation. Briefly, PEGylated-chitosan was dissolved in double distilled water (1.0% w/v; 1 mL). Then, the polymer solution was added to 1 mL of methylene chloride containing 20% (w/v) poly vinyl alcohol as surfactant and sonicated for 1 min over ice bath. Subsequently, the emulsion was stirred and 5 mL of aqueous genipin solution (0.05 mM) was added drop-wise to the previously prepared emulsion. The emulsion was stirred (1000 rpm) at room temperature for ~12 h to crosslink the nanoparticles and evaporation of methylene chloride. Drug-loaded nanoparticles were prepared by dissolving predetermined amount of drug in aqueous polymer solution, and the synthesis procedure was performed as described above. Crosslinked nanoparticles were then separated from the solution by ultracentrifugation (SIGMA 3–16 K Centrifuge, UK) at 20,000 g and were washed twice with DI water to remove excess unreacted polymer, crosslinker, and the free drug. Finally, nanoparticles were freeze dried and stored at 4 °C until further use.

### Nanoparticle Characterization

Nanoparticle size was determined by three different methods including AFM in the tapping mode, DLS, and TEM. Silicon tapping tips (TESP, VEECO) were used to perform AFM (JPK, Nanowizard 2, JPK Instruments, Germany). Samples were prepared by dispersing nanoparticles in DI water at 1 mg/ml concentration and placing one drop on the polyethylene imine coated glass cover slip then air dried. Samples imaging was conducted by using Nano-scope III (Digital Instrument/VEECO). TEM imaging of nanoparticles was performed following negative staining with uranyl acetate. Samples were prepared similar to AFM test, but were placed on formvar-coated copper grids (Ted Pella, Inc., Redding, CA) and were tested using a TEM instrument (TEM, Philips/FEI, Inc., NY). The diameters of about fifty nanoparticles in the TEM images were calculated by using a digital caliper manually. The nanoparticle size was also measured by DLS using a Malvern Zetasizer Nano ZS (Malvern Instruments, UK) at a fixed scattering angle (90°).

### Drug loading and encapsulation efficiency

The drug loading (DL, w/w%) of nanoparticles was measure by dissolving freeze dried nanoparticles in water followed by determining their drug content using a HPLC system (Agilent Technologies Inc, 1200, CA, USA) equipped with a UV detector (Agilent Technologies Inc, 1200) and reversed phase column (ODS C18, 5 µm, 4.6 mm × 250 mm, Dikma, China). The mobile phase contained a mixture of acetonitrile and monobasic sodium phosphate buffer with the ratio of 17:83 (v/v%) with a flow rate of 1 mL/min. The signal was measured at 254 nm to determine the drug concentration. The DL of the nanoparticles was then calculated^44^:

1 $${DL} \% =\frac{{\rm{Total}}\,{\rm{mass}}\,{\rm{of}}\,{\rm{drug}}\,{\rm{extracted}}\,{\rm{from}}\,{\rm{freeze}}\,{\rm{dried}}\,{\rm{nanoparticles}}}{{\rm{Total}}\,{\rm{mass}}\,{\rm{of}}\,{\rm{freeze}}\,{\rm{dried}}\,{\rm{nanoparticles}}}\times 100$$

The concentration of drug in the aqueous phase was determined using HPLC as described previously. Encapsulation efficiency (EE) was reported as:

2 $${EE} \% =\frac{{\rm{Total}}\,{\rm{amount}}\,{\rm{of}}\,{\rm{drug}}\,{\rm{inside}}\,{\rm{nanoparticles}}}{{\rm{Total}}\,{\rm{amount}}\,{\rm{of}}\,{\rm{drug}}\,{\rm{in}}\,{\rm{the}}\,{\rm{initial}}\,{\rm{solution}}}\times 100$$

It should be noted that EE shows the ratio of the actual encapsulated drug over the intended value, while DL is referring to the actual encapsulated drug quantity with respect to the dry weight of nanoparticles.

### Drug release from nanofibrous meshes with integrated heaters

The device with the heater was placed on the surface of 1.5 ml of freshly prepared PBS and a tiny magnetic stirrer bar was placed in PBS media, and different electrical voltage was applied to the conductive pattern to generate heat. The drug released during different voltages, temperature and time was measured by HPLC based on the method previously mentioned^45, 46^. The surface temperature of the device measured by Welch Allyn 105801 Caretemp Touch Free Infrared Thermometer. According to the manufacturer information the accuracy of the system is ±0.5 °C.

### Electrospinning

PGS and PCL were dissolved at ratios ranging from 1:1–1:5 in anhydrous chloroform: ethanol (9:1, v/v) mixture, where the total polymer concentration was kept constant at 20% (w/v). An electrical voltage of 19.5 kV over a distance of 15 cm was applied and the prepolymer mixture flow rate was set at 1.5 mL/h during electrospinning. The prepolymer mixture was spun to obtain a nanofibrous sheet (~170 μm thickness). The electrospun sheet was dried using a fan for 12 h with airflow rate of 12 m^3^ h^−1^ to remove any residual solvent prior to use. Nanoparticles-loaded nanofibers were prepared by dissolving predetermined amount of nanoparticles in pre polymer solution, ultrasound to make it homogeneous and the electrospinning was performed as mentioned above.

### Degradation

Patterned electrospun sheets were placed in polystyrene Petri dishes containing 2.5 × 10^−3^ M of NaOH in PBS solution (5 mL). Samples were kept in NaOH solution at 37 °C. A digital camera was used to capture the photographs of the nanofibrous sheets.

### Scanning Electron Microscopy (SEM)

Dried electrospun samples were mounted on aluminum stubs using copper adhesive tape. The SEM images of the gold coated samples were acquired using a JEOL JSM 7600 F (10 KV) to determine the microarchitecture of the electrospun nanofibrous sheets.

### X-ray Diffraction (XRD)

X-ray diffraction data of the coated sheets were acquired using Ultima-IV diffractometer (Rigaku, Japan) (Cu Kα radiation *λ* = 1.5418 Å at 40 kV accelerating voltage and 30 mA) with parallel beam geometry and a multipurpose thin film attachment. For all nanofibrous sheets, the XRD patterns were recorded in theta-2theta, grazing incidence angles of 4° to 8°, 0.05° increment size and 2 s count time per step.

### Electrically conductive pattern fabrication and testing

Conductive patterns were fabricated from Zn/Mg/Ag using radio frequency (RF) magnetron sputtering (DC/RF Magnetron Sputter System, Syskey Technologies, Taiwan). Electrospun sheets were covered with a shadow mask, which was prepared by cutting a transparent sticker sheet using a laser engraver (Versalaser, VLS2.30). High purity targets of Zn/Mg/Ag (3.0 × 0.6 in) were used. To prepare nanocrystalline Zn/Mg/Ag thin films, plasma was generated inside a chamber using argon gas with a flow rate of 20 sccm at RF power of 100 W while the base pressure and operating pressure were 1 × 10^−6^ and 5 × 10^−3^ torr. The substrate rotation, target-substrate distance and deposition time were 15 rpm, 14 cm and 1000 s, respectively with an approximately 500 nm film thickness that was measured using a surface profiler (DektakXT, Bruker, Germany). The thickness of the film was controlled by increasing or decreasing the deposition time. Finally, the shadow masks were removed from the sheets leaving the patterned electrical circuit on the electrospun sheets. The coated sheets were subsequently used for electrical conductivity measurements using a multimeter.

### Bacterial study

A wide range of bacterial strains were used for the experiments to verify the effectiveness of the release antibiotics: *Escherichia coli, Pseudomonas fluorescens, Salmonella typhimurium, Vibrio parahaemolyticus, Listeria monocytogenes, Bacillus megaterium, Bacillus cereus, Staphylococcus aureus, Lactobacillus plantarum, Lactobacillus brevis, and Lactobacillus bulgaricus*. The bacteria were inoculated and incubated in an air bath shaker (37 °C, 130 rpm) for 12 h until the culture entered the exponential period of growth. Bacteria with the concentration of ~1 × 10^5^ CFU/mL were cultured for zone of inhibition and colony forming unit counting experiments. To investigate the application of device on bacterial, the device was placed on the surface of cultured bacteria, and different electrical voltages were applied to the conductive pattern to generate heat. A blank control sample without antibiotics was prepared for comparison. All the plates were incubated at approximately 34 °C for an appropriate time. Finally, the plates were taken out and the inhibition zone and number of remaining colonies were calculated.

### Cell studies

Keratinocytes were cultured in a DMEM-based medium containing 10% FBS and 1% penicillin/streptomycin. For cytotoxicity assessment, circular samples (1 cm in diameter) were prepared and placed at the bottom of 24-well polystyrene plates. Then, cells were resuspened in culture media at the concentration of 1 × 10^7^ cells/mL and 5,000 cells were seeded on the samples. The samples were incubated for 1 hr to allow cells to attach and then 300 µL of culture medium was added to each well. Cellular metabolic activity was measured using PrestoBlue® assay following manufacturer protocol on days 1, 3 and 7 and the assay results were measured using a BioTek UV/vis Synnergy 2 microplate reader.

### Statistical analysis

Data from continuous parameters are expressed as means ± standard deviation (SD) of measurements (*p < 0.05, **p < 0.01 and ***p < 0.001) and were compared using one-way ANOVA testing in GraphPad Prism 6.

## Electronic supplementary material

Supplementary information
