# Supplementary material for: Biodegradable elastic nanofibrous platforms with integrated flexible heaters for on-demand drug delivery
Source: Sci Rep. 2017 Aug 23;7:9220. doi: 10.1038/s41598-017-04749-8 (PMC5569034; doi:10.1038/s41598-017-04749-8)
Supplement: Supplementary file 1 — Supplementary information [file 41598_2017_4749_MOESM1_ESM.pdf]

SUPPORTING INFORMATION

Title. Biodegradable elastic nanofibrous platforms with integrated flexible heaters for on-demand drug delivery

Authors. Ali Tamayol[1,2,3]*, Alireza Hassani Najafabadi[1,2,4]*, Pooria Mostafalu[1,2,3], Ali K. Yetisen[5], Mattia Commotto[1,2], Musab Aldhahri[6,7], Mohamed Shaaban Abdel-wahab[6], Zeynab Izadi Najafabadi[8], Shahrzad Latifi[9], Mohsen Akbari[1,2,3,10], Nasim Annabi[1,2,11], Seok Hyun Yun[4], Adnan Memic[6], Mehmet R. Dokmeci[1,2,3], Ali Khademhosseini[1,2,3,6,12,13]**

Mechanical characteristics of nanofibrous mesh:

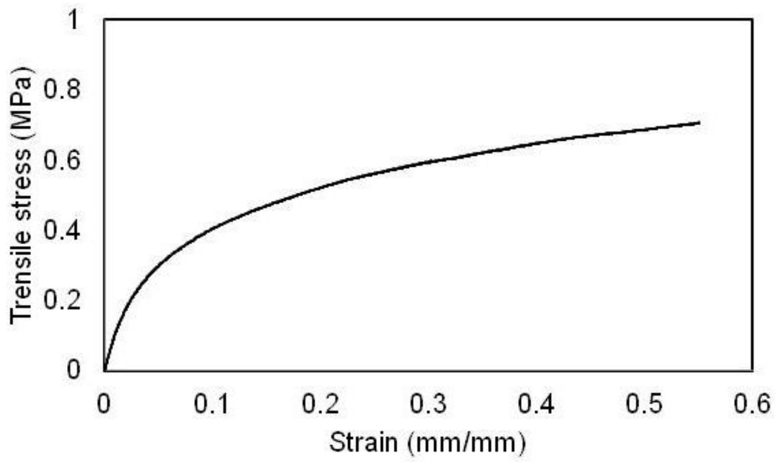

Figure S1. A typical stress-strain curve of PGS-PCL nanofibrous meshes.

Characterization of PEGylated-chitosan:

The FTIR spectrum of native chitosan and chitosan–PEG showed in Fig S2. The FTIR spectrum of native chitosan illustrated a broadened peak at 3200–3600 cm^{-1} which can be related to -OH and -NH stretching vibration. However, the FTIR spectra of the chitosan–PEG showed a slightly shifted in of hydroxyl group of chitosan that can be related to PEGylation of the hydroxyl group. In addition,the appearance of new bands at 1024 (C-O), 1280, 1320, 1480, 870 and 3200–3600 (CH2,O-CH2) is again can be attributed to the reaction of the hydroxyl groups of chitosan with PEG (FTIR spectrum of native mPEG showed peaks at 2890 cm^{-1} (C-H stretching), 1100 (C-O stretching), 1474 and 1278 cm^{-1}). Therefore, it is supposed that mPEG was successfully conjugated to hydroxyl group of chitosan.

The NMR data of the chitosan–PEG indicated the sharp peak of PEG–OCH3 around 3.31 ppm, and strong signal around 3.5–3.8 ppm (PEG–OCH2CH2O) and 2.3 ppm (OCOCH2). Also, in the chitosan–PEG spectrum, signals in the range of 3.6–4.0 ppm were not well distinguishable since the PEG methylene peak with the peaks from the chitosan skeleton. overlap to each other , but, the peak at 2.85 ppm can be used to investigate the H-2 unsubstituted glucosamine residue in the chitosan.

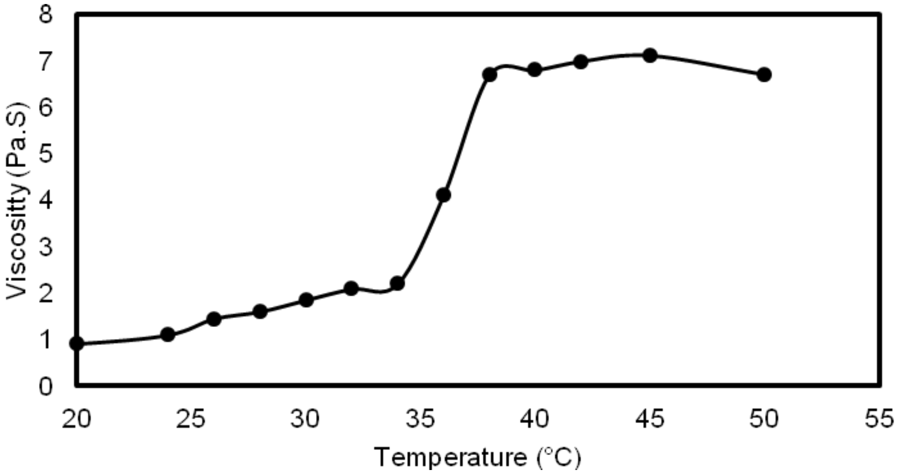

Figure S2. Rheological properties of PEGylated-chitosan demonstrating the change in its response around its critical temperature of 37 °C.

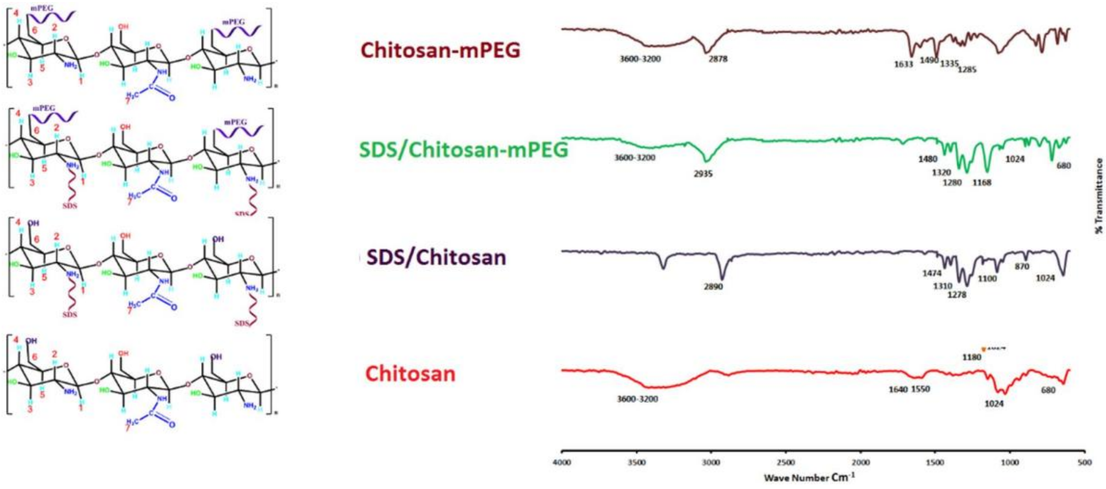

Figure S3. Synthesis of the thermoresponsive nanoparticles. FTIR of proposed polymer for preparing thermo sensitive nanoparticles.

Characterization of the fabricated nanoparticles:

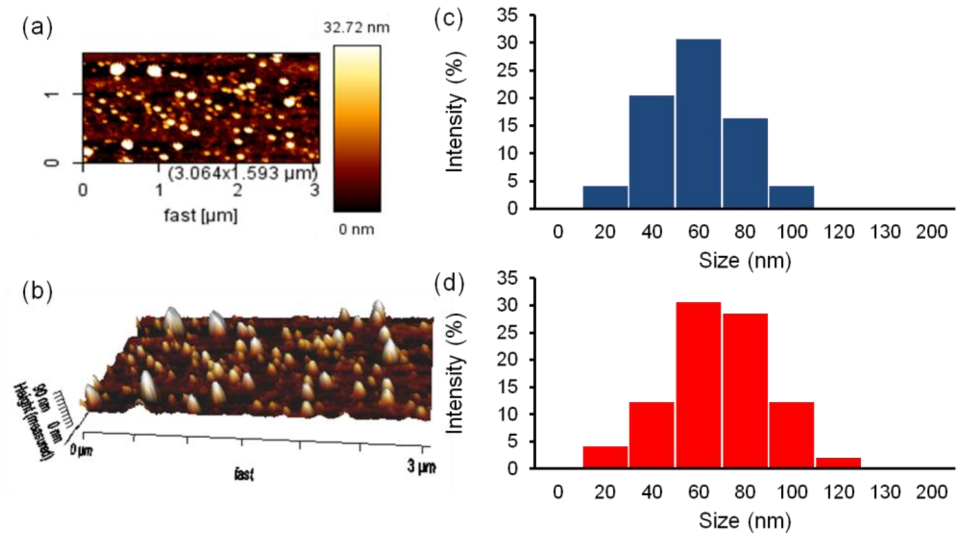

Figure S4. Characterization of the size of the fabricated nanoparticles. (a,b) AFM images of the engineered nanoparticles. (c) Size distribution of the fabricated particles measured from TEM images. (d) Size distribution of the fabricated particles measured from AFM images.

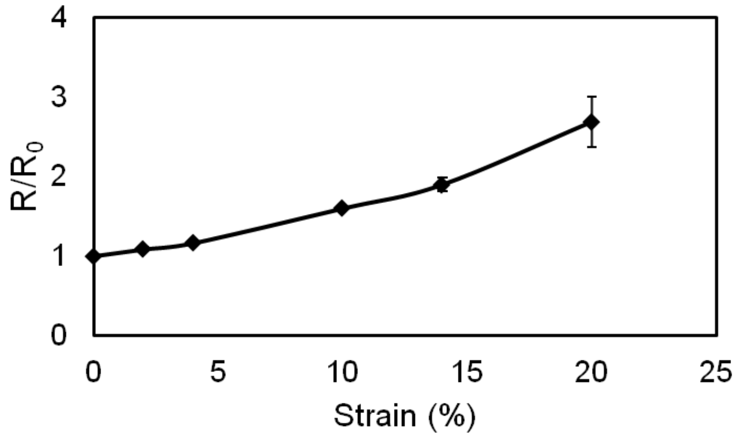

Figure S5. Effect of strain on the electrical resistance of a typical conductive pattern. The electrical resistance values at various strain rates (R) are normalized with respect to values at the under non stretch state (R_0).

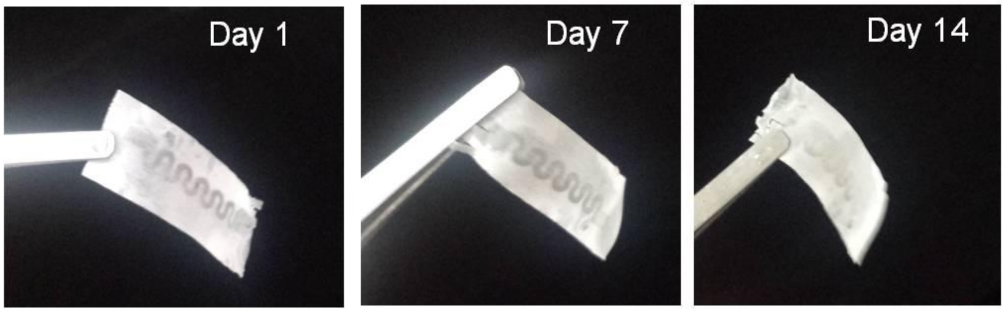

Figure S6. Degradation of zinc patterns in aqueous solution with pH 8.5.

Tables

Table S1. Effect of added cefazolin on drug encapsulation, concentration of PEG-chitosan was 1.0 (%w/v).

Amount (%)	Encapsulation Efficiency (%)
0.1	99.1±0.12
0.5	98.6±0.9
1.5	91.4±1.4
2	85.3±1.9
3	83.3±1.3
3.5	75.2±1.1

Table S2. Effect of added ceftriaxone on drug encapsulation, concentration of PEG-chitosan was 1.0 (%w/v).

Amount (%)	Encapsulation Efficiency (%)
0.1	94.1±0.26
0.5	93.1±0.85
1.5	91.2±1.5
2	91.3±2.7
3	91.1±1.7
3.5	68.3±1.8

Table S3. Drug loading characteristics of PEGylated-chitosan nanoparticles (means ±S.D, n=5). Drug loadings of cefazolin and ceftriaxone are 3±0.01% (%w/w)

Concentration of PEG-chitosan (%w/v)	Encapsulation efficiency of cefazolin (%)	Encapsulation efficiency of ceftriaxone (%)
0.3	49.2±2.1	54.2±1.9
0.5	78.1±1.8	80.4±1.3
0.7	81.6±1.4	87.1±2.6
1	83.3±1.3	91.1±1.7

Table S4. The effect of applied heat on the system immersed in bacterial culture on the survival of 11 different bacteria (log CFU/mL).

**Bacteria**		**Initial concentration**	**Control after 24 hr**	**Samples heated at 38 °C after 24 hr**
Gram (-)	Escherichia coli	7.92±0.5	9.59±0.5	1.98±0.1
	Pseudomonas fluorescens	6.64±0.3	8.79 ±0.7	1.78±0.08
	Salmonella typhimurium	7.54±0.5	9.62 ±0.4	1.54±0.03
	Vibrio parahaemolyticus	6.36±0.2	8.51 ±0.4	1.41±0.01
Gram (+)	Listeria monocytogenes	6.24±0.7	8.19±0.2	1.31±0.01
	Bacillus megaterium	6.72±0.2	9.97±0.1	1.77±0.01
	Bacillus cereus	5.36±0.5	7.18±0.6	1.52±0.04
	Staphylococcus aureus	5.78±0.1	7.34±0.2	2.02±0.07
	Lactobacillus plantarum	5.21±0.2	7.97±0.3	1.78±0.06
	Lactobacillus brevis	6.32±0.1	9.19±0.2	1.88±0.01
	Lactobacillus bulgaricus	6.83±0.8	9.07±0.9	1.78±0.08
